# Large‐scale assessment of genetic structure to assess risk of populations of a large herbivore to disease

**DOI:** 10.1002/ece3.11347

**Published:** 2024-05-20

**Authors:** W. David Walter, Alberto Fameli, Kelly Russo‐Petrick, Jessie E. Edson, Christopher S. Rosenberry, Krysten L. Schuler, Michael J. Tonkovich

**Affiliations:** ^1^ U.S. Geological Survey, Pennsylvania Cooperative Fish and Wildlife Research Unit The Pennsylvania State University University Park Pennsylvania USA; ^2^ Pennsylvania Cooperative Fish and Wildlife Research Unit The Pennsylvania State University University Park Pennsylvania USA; ^3^ Pennsylvania Game Commission Harrisburg Pennsylvania USA; ^4^ Cornell Wildlife Health Lab, New York State Wildlife Health Program Ithaca New York USA; ^5^ Ohio Department of Natural Resources, Division of Wildlife Athens Ohio USA

**Keywords:** chronic wasting disease, genetic structure, large‐scale, population assignment, population structure, white‐tailed deer

## Abstract

Chronic wasting disease (CWD) can spread among cervids by direct and indirect transmission, the former being more likely in emerging areas. Identifying subpopulations allows the delineation of focal areas to target for intervention. We aimed to assess the population structure of white‐tailed deer (*Odocoileus virginianus*) in the northeastern United States at a regional scale to inform managers regarding gene flow throughout the region. We genotyped 10 microsatellites in 5701 wild deer samples from Maryland, New York, Ohio, Pennsylvania, and Virginia. We evaluated the distribution of genetic variability through spatial principal component analysis and inferred genetic structure using non‐spatial and spatial Bayesian clustering algorithms (BCAs). We simulated populations representing each inferred wild cluster, wild deer in each state and each physiographic province, total wild population, and a captive population. We conducted genetic assignment tests using these potential sources, calculating the probability of samples being correctly assigned to their origin. Non‐spatial BCA identified two clusters across the region, while spatial BCA suggested a maximum of nine clusters. Assignment tests correctly placed deer into captive or wild origin in most cases (94%), as previously reported, but performance varied when assigning wild deer to more specific origins. Assignments to clusters inferred via non‐spatial BCA performed well, but efficiency was greatly reduced when assigning samples to clusters inferred via spatial BCA. Differences between spatial BCA clusters are not strong enough to make assignment tests a reliable method for inferring the geographic origin of deer using 10 microsatellites. However, the genetic distinction between clusters may indicate natural and anthropogenic barriers of interest for management.

## Graphical abstract

Chronic wasting disease (CWD) can spread among cervids by direct and indirect transmission, the former being more likely in emerging areas. Identifying subpopulations allows the delineation of focal areas to target for intervention. Differences between spatial Bayesian clustering algorithms clusters are not strong enough to make assignment tests a reliable method for inferring the geographic origin of deer using 10 microsatellites, however, genetic distinction between clusters may indicate natural and anthropogenic barriers of interest for management.

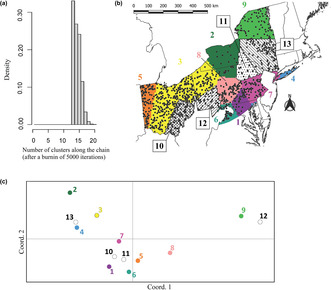

## 1 INTRODUCTION

Disease is a major cause of population decline or extinction for many wildlife species (McKnight et al., 2017; Palkovacs et al., 2004; Sutherland et al., 2018). Population fluctuations from disease can also rapidly and drastically decrease genetic diversity, which results in less resilient populations (Haworth et al., 2021; Mathews & Porter, 1993; Phillips et al., 2020). Identifying the factors underlying disease spread is important to prevent and manage disease more effectively; however, current management is typically based on geopolitical or geographic boundaries (Rosenberry & Diefenbach, 2019) rather than biological or landscape processes.

Population genetic structure is typically correlated with the spread of disease, as both disease transmission and gene flow are influenced by the movement of individuals between populations and subpopulations (Kelly et al., 2010; Saunders et al., 2012). Disease can also decrease genetic connectivity between populations, with disease‐affected populations being more genetically different from unaffected populations (Addison & Hart, 2004). Identifying discontinuities among subpopulations through indices of gene flow can allow for the delineation of biologically appropriate management units (Moritz, 1994) and identification of areas with high risk of disease spread to target for effective intervention (Holderegger & Wagner, 2006; Rees et al., 2008; Storfer et al., 2010). Therefore, studying population genetic structure and connectivity could help predict future disease spread and improve prevention and mitigation efforts.

One disease in need of study through assessment of population structure is chronic wasting disease (CWD), a transmissible spongiform encephalopathy affecting members of the Cervidae family, caused by a misfolded isoform of the prion protein PrP^C^ (Cullingham, Merrill, et al., 2011; Miller & Williams, 2004; Williams et al., 2002). The first case of CWD was detected in 1967 in captive mule deer (*Odocoileus hemionus*) in Colorado (Williams & Young, 1980) and it has now been detected in 33 US states (USGS, 2024), Canada (Kahn et al., 2004), South Korea (Sohn et al., 2002), and Scandinavia (Vikøren et al., 2019).

Chronic wasting disease spreads through direct and indirect interactions between individuals even before symptoms occur (Sigurdson, 2008). Symptoms include weight loss and behavioral changes (Williams, 2005), but they do not appear until at least a year after exposure (Williams et al., 2002). Since CWD has no barriers to transmission and a long infectious period, its prevalence can be up to 50% in wild populations and even higher in captive populations (Miller & Williams, 2004; O'Rourke et al., 2004). While CWD prevalence is likely underestimated because of constraints on surveillance, it appears to have increased over time (Escobar et al., 2020; Haworth et al., 2021; Schuler et al., 2018). The disease is fatal and there is currently no treatment (Miller & Williams, 2004). If left unchecked, models predict CWD will lead to long‐term population declines of multiple species and possibly local extinction (Edmunds et al., 2016; Miller & Williams, 2004). Chronic wasting disease is difficult to eliminate from wild populations since the amount of infectious prions in the environment increases with the number of infectious animals (Gross & Miller, 2001; Manjerovic et al., 2014). Therefore, management tends to be less effective the longer the disease has progressed, and adequate data on underlying factors are important for implementing effective management (Hanley et al., 2022).

White‐tailed deer (*Odocoileus virginianus*) serves as an ideal species for studying factors related to CWD, given its status as the most common host and widespread distribution among affected species (Heffelfinger, 2011; Miller, Miller‐Butterworth, et al., 2020). White‐tailed deer are also a critically important species to human culture and the economy in North America, in addition to filling important ecological roles as herbivores and prey (Bishop, 2004; Haworth et al., 2021; McShea, 2012). Chronic wasting disease prevalence increases in subpopulations with higher connectivity and declines farther from these affected areas (Blanchong et al., 2008; Conner & Miller, 2004; Evans et al., 2016). Since white‐tailed deer are broadly distributed habitat generalists, defining boundaries between subpopulations can be difficult when structure exists, although genetic clustering algorithms can aid in this process (Miller, Cullingham, et al., 2020; Vergara et al., 2015). Due to the difficulty in determining these boundaries and the importance of connectivity to the spread of CWD, further study of connectivity and genetic structure will be critical to efforts to manage CWD.

Multiple studies have concluded that gene flow tends to be widespread for white‐tailed deer (Bauder et al., 2021; Kelly et al., 2010; Miller, Miller‐Butterworth, et al., 2020; Robinson et al., 2012). Landscape structures such as interstate highways and rivers provide some impediment to genetic connectivity, leading to population structuring (Blanchong et al., 2008; Kelly et al., 2010). For example, 11 genetic clusters that were divided into 4–5 subpopulations based on ecophysiographic provinces were identified in parts of Pennsylvania, Maryland, and Virginia, United States, where CWD is present using spatially explicit Bayesian clustering methods (Miller, Miller‐Butterworth, et al., 2020). Four clusters also were found in CWD‐affected areas including part of Illinois and Wisconsin (Kelly et al., 2010).

Genetic research can also be applied to determine the origin of infected deer, for example, the identification of a deer of wild origin in a captive facility that was later identified as a rescued orphan fawn from the wild in the Mid‐Atlantic region, United States (Miller & Walter, 2020). This methodology can go a step further and provide insights about the geographic origin of wild animals, with numerous examples in the literature describing its application to assist law enforcement and management of other species. For example, genetic assignments have been used to infer the geographic origin of trafficked animals (Dominguez et al., 2019; Ogden & Linacre, 2015; Oklander et al., 2020), invasive species (Caldera et al., 2008; Russell et al., 2010; Signorile et al., 2016) or migrants (Buchalski et al., 2015; Gajdárová et al., 2021). A prerequisite for conducting these assignment methods is to characterize potential sources of individuals in terms of their allele frequencies, as there need to be significant differences between sources to achieve correct assignments (Araujo et al., 2014; Nyce et al., 2013; Rosel et al., 2017). Providing wildlife managers with information about the most likely geographic origin of CWD‐infected deer could allow more effective control actions by incorporating source‐sink dynamics, detecting anthropogenic movement of deer, and evaluating whether a CWD detection could constitute a sign of local establishment of the disease.

Our objectives were (1) to assess white‐tailed deer genetic structure across the Mid‐Atlantic region of the United States, to detect genetic units that could be incorporated into management actions, and (2) to evaluate the effectiveness of genetic assignment tests to correctly match deer to their origin. Previous studies of genetic structure in white‐tailed deer were limited in extent, typically only including data from a single state or portions of a few states, for example, Ohio (Bauder et al., 2021), south‐central Wisconsin (Blanchong et al., 2008), Arkansas (Chafin et al., 2021), part of Illinois (Kelly et al., 2010), the area surrounding the border between Iowa and Wisconsin (Lang & Blanchong, 2012) or between Illinois and Wisconsin (Robinson et al., 2012). Given the widespread distribution of white‐tailed deer, fostering collaboration between state management agencies that are potentially genetically linked by the gene flow of deer could aid in the management of CWD. Therefore, this study covers a much wider area and includes more samples than previous research on white‐tailed deer population genetics in the region, with the previous largest covering portions of three states (Miller, Miller‐Butterworth, et al., 2020). Genetic assignment tests have been successfully applied when assigning deer to either a captive or wild population in central Pennsylvania, Maryland, and Virginia (Miller & Walter, 2020). We expanded this work by including samples from Ohio, New York, and statewide in Pennsylvania, as well as using our inferences of genetic structure in the wild population to gain geographic resolution in our assignment tests. We included the same samples from captive deer used by Miller and Walter (2020) and used the same genetic assignment tests to assess whether the high efficiency in assignments reported by these authors was maintained after expanding the representation of the wild population, and after subdividing the wild population into genetic clusters. Finally, we compared the performance of these assignments based on genetic clusters with control assignments based on anthropogenic boundaries (i.e. states and physiographic provinces) for which we expected low efficiency given that these boundaries may not encompass genetic structure.

## 2 METHODS

### 2.1 Sample collection and study area

**FIGURE 1:**
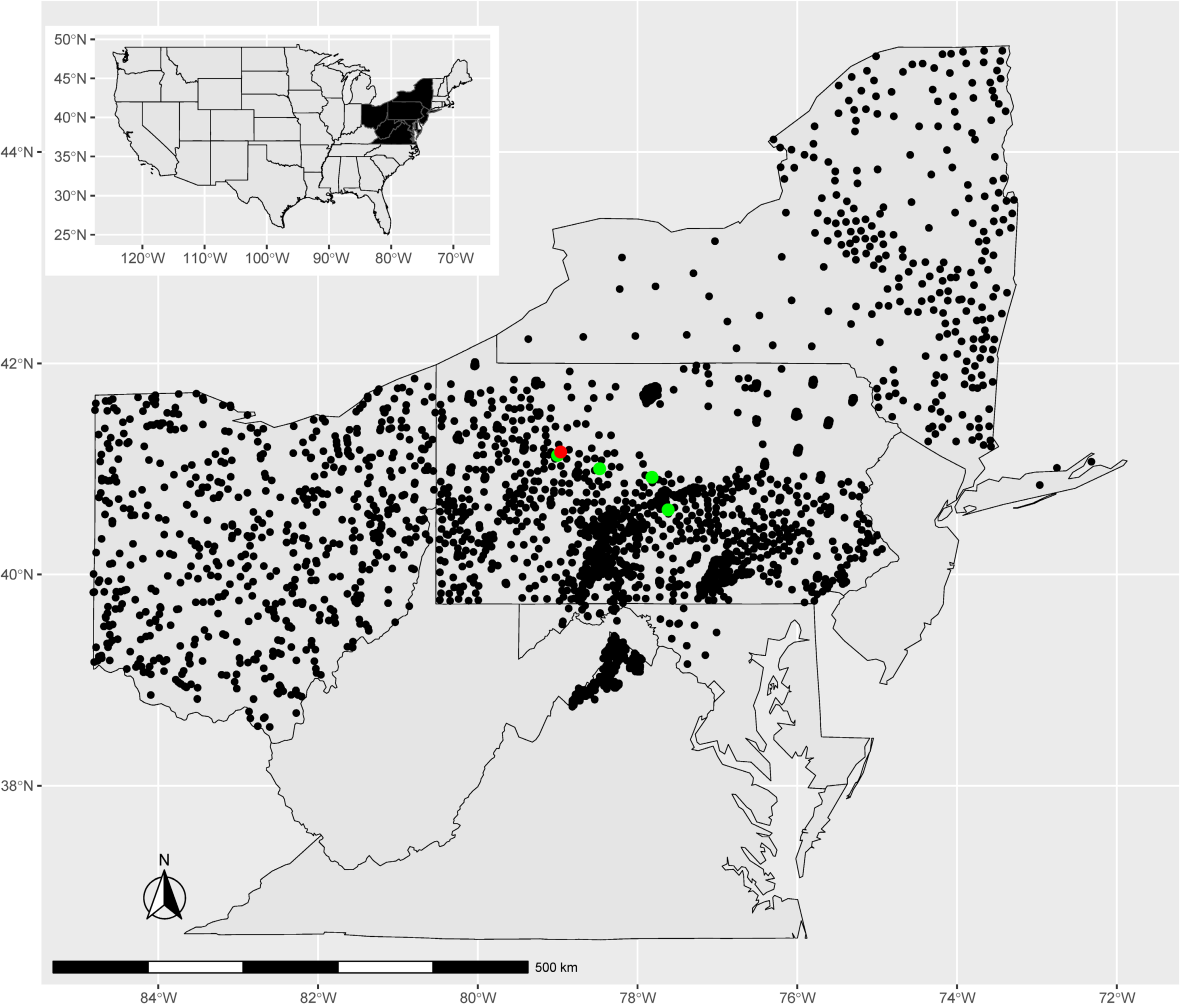
Map of our study area, with black symbols representing all samples collected from wild white‐tailed deer (*Odocoileus virginianus*, *n* = 5701) in the mid‐Atlantic region of the United States, including the states of Ohio, Pennsylvania, New York, part of Maryland and part of Virginia. Locations of four captive cervid facilities where no disease was detected (green) and one previously infected facility (red) were mapped to county centroids in order to maintain anonymity. We included 50 samples from these facilities in our study, used in a previous study applying genetic assignments (Miller & Walter, 2020).

We included a total of 5701 samples from various tissues of wild white‐tailed deer, obtained from hunter‐harvested individuals, roadkill, deer collected for other research studies, and individuals obtained through routine disease surveillance by collaborating management agencies from 2014 to 2022 (Miller et al., 2019; Walter et al., 2023). We collected geographic coordinates of deer sample locations when possible, otherwise, we assigned samples to the centroid of the county of origin, or township when available. These samples represented part of Maryland (*n* = 138), part of Virginia (*n* = 281), and most of New York (*n* = 626), Pennsylvania (*n* = 4013), and Ohio (*n* = 643) (Figure 1). Chronic wasting disease has previously been recorded in all the states sampled, although it is not currently detected in wild deer in New York (Evans et al., 2014; USGS, 2024). Samples represented 215 counties, with *n* = 1–325 per county. Our study area covered multiple physiographic provinces, as defined by Fenneman (1928): Valley and Ridge (*n* = 2,082), St. Lawrence Valley (*n* = 58), Piedmont (*n* = 491), New England (*n* = 40), Interior Low Plateau (*n* = 4), Coastal Plain (*n* = 17), Central Lowland (*n* = 374), Blue Ridge (*n* = 62), Appalachian Plateaus (*n* = 2484), and Adirondack (*n* = 89).

Samples from captive deer (*n* = 50) were obtained from five facilities in central Pennsylvania. One of these facilities was infected with CWD and the other four did not have CWD detected at the time of sample collection. Detailed information regarding sample collection from captive deer can be found in Miller and Walter (2020).

### 2.2 DNA extraction and microsatellite genotyping

We performed DNA extractions using QIAGEN DNeasy blood and tissue extraction kits (QIAGEN, Valencia, CA, USA) following the protocol outlined in Miller et al. (2019) for samples from Maryland, Virginia, New York and some of the samples from Pennsylvania (collected between 2014 and 2019). The rest of the samples from Pennsylvania (collected in 2020 and 2021) and Ohio samples were extracted using a QIAmp 96 DNA QIAcube HT extraction kit following the manufacturer's recommendations. We genotyped all samples for 11 microsatellite loci that were previously validated and provided in a protocol for this region (Miller et al., 2019). We removed locus OvirQ because it showed a pattern of alleles in our dataset that was inconsistent with the reported motif, with some alleles differing from others only by one base pair, making genotyping unreliable. Therefore, we evaluated 10 loci for all analyses presented.

### 2.3 Genetic variability

We conducted a spatial principal component analysis (sPCA) to evaluate the distribution of genetic variability in our study area, using the package *adegenet* in program R v.4.2.0 (Jombart et al., 2008; R Core Team, 2022). This multivariate method is designed to identify spatial genetic patterns: global structures when genetic autocorrelation is positive (e.g. isolation by distance phenomenon or presence of genetic clusters) or local structures when genetic autocorrelation is negative (i.e. when neighboring sites tend to be dissimilar). This analysis requires a user‐defined connectivity network, but unlike Bayesian clustering algorithms, it does not assume Hardy–Weinberg (H–W) equilibrium or linkage equilibrium (Jombart et al., 2008). Eigenvalues of sPCA are composite, representing both genetic variability and spatial autocorrelation, instead of representing only genetic variability comparable to a classical principal component analysis. We used all samples from wild deer (*n* = 5701) and two connectivity networks: (a) setting the maximum distance between individuals so that each site had at least one connection (hereafter referred to as “distance‐based network”), and (b) Delaunay triangulation which required noise to be added to coordinates (we used a noise of 5 km which we considered small enough given that our study area spans about 1028 km east–west and 845 km north–south). In addition to the visual interpretation of the eigenvalues bar plot, we conducted a statistical test to evaluate the presence of global or local structures (i.e. positive or negative spatial autocorrelation, respectively), with 999 and 9999 permutations and *α* = .01 (Montano & Jombart, 2017). We created a screeplot showing the decomposition of eigenvalues into spatial autocorrelation and variance captured, in order to decide which principal components to interpret. All further analyses, unless stated otherwise, were conducted in program R v.4.2.2.

### 2.4 Population structure

#### 2.4.1 Non‐spatial Bayesian clustering algorithm

We evaluated the presence of genetic clusters in our dataset (i.e. demographic units with different allele frequencies due to limited gene flow among them) using a non‐spatial Bayesian clustering algorithm (BCA). We included all wild deer samples (*n* = 5701) and used a burn‐in period of 500,000 iterations followed by 1000,000 iterations in STRUCTURE 2.3.4 (Pritchard et al., 2000). We assumed 1–10 clusters (*K*) and performed 10 independent runs per *K* using an admixture model and correlated allele frequencies, with all remaining parameters on default settings. Given that our samples were unevenly distributed, we subsampled deer and reran the test, comparing these results to the ones obtained for the full dataset and confirming accuracy by reducing sampling bias. These subsamples consisted of a maximum of one, two, and five deer per sex per county (*n* = 399, 750, and 1557, respectively). We ran STRUCTURE with these subsamples and used the settings described for the whole dataset of wild deer. We summarized results in STRUCTURE Harvester (Earl & vonHoldt, 2012) and determined the number of genetic clusters that better represented our data based on Pr(*X*|*K*) (i.e. the mean log probability of data, Pritchard et al., 2000) and Evanno's ∆*K* method (Evanno et al., 2005). Following a relatively conservative approach, we considered individuals as being part of a genetic cluster only if their membership value was 0.7 or more (Cullingham, Nakada, et al., 2011; Lang & Blanchong, 2012). We generated membership bar plots averaging results from independent runs performed for each *K* value and assessed the convergence of these runs in CLUMPAK v.1.1.2 (Kopelman et al., 2015). We could not use CLUMPAK for the full wild deer dataset because the number of samples exceeded the software's limit, so we used CLUMPP 1.1.2 instead (Jakobsson & Rosenberg, 2007).

Given the large area covered in our study, we were concerned about possible isolation by distance (IBD) patterns in genetic variability creating spurious clusters (Frantz et al., 2009; Schwartz & McKelvey, 2009). To evaluate this possibility, we reran STRUCTURE with our subsample of two deer per sex per county but excluding samples from extreme west and east locations to retain about 60% of the 750 individuals in the subsample. This reduced dataset of two deer per sex per county without extreme west and east locations was comprised of 461 samples. We assumed that such a drastic reduction in geographic coverage would result in differences in cluster detection and/or their geographic boundaries if the underlying IBD pattern was influencing non‐spatial BCA results. We performed a Mantel test using GenAlEx (Peakall & Smouse, 2006, 2012) with 999 permutations and an *α* = .01, to assess the correlation between geographic and genetic distance in our subsample of two deer per sex per county without extreme west and east locations (*n* = 461).

#### 2.4.2 Spatial Bayesian clustering algorithm

We also evaluated the presence of genetic clusters in our dataset by conducting a spatial BCA, which incorporates geographic coordinates of the samples as priors, using GeneLand (Guedj & Guillot, 2011; Guillot, 2008; Guillot et al., 2008; Guillot, Estoup, et al., 2005; Guillot, Mortier, et al., 2005; Guillot & Santos, 2010). This method can outperform non‐spatial BCA and detect low levels of population differentiation (Guillot, Mortier, & Estoup, 2005) and its usage has been recommended in cases where there could be IBD (Perez et al., 2018). It should be noted that spatial BCA can overestimate the number of clusters (Chen et al., 2007); therefore, it is useful to run both non‐spatial and spatial BCA as these methods could retrieve different levels of hierarchical population structure. Based on the results obtained with non‐spatial BCA, we decided to conduct this spatial BCA using our subsets of two and five deer per sex per county (*n* = 750 and 1557 respectively). Due to computational constraints and to reduce the effects of sampling bias, we did not perform this analysis using the full wild deer dataset. As recommended by the developers, we set the maximum rate of the Poisson process equal to the number of individuals (i.e. 750 or 1557) and the maximum number of nuclei equal to number of individuals times three (i.e. 2250 or 4671). We run the models for *K* = 1–20 (number of clusters), 2 million iterations, thinning = 100, allele frequencies correlated, spatial model = TRUE, null allele model = TRUE, and multiple independent runs = 10. Because some samples lacked collection site coordinates and were assigned to the centroid of their county of origin, we followed Bauder et al. (2021) to include uncertainty on geographic position. For this uncertainty, we calculated the average county area in our study and obtained its square root, which represents the edge length of a square equal in area to the average county. We used this value as our uncertainty term on geographic position (40,743 m). We selected the independent run that had the highest mean log posterior density to infer the number of clusters that best represented our data. We performed post‐processing of this run with a burn‐in of 5000, setting the number of pixels for map interpolation to 100 for the *x*‐axis and 100 for the *y*‐axis. We retrieved summary statistics, evaluated the proper mixing of the iterations, and generated a map of the spatial distribution of genetic clusters. We assessed the similarity between inferred clusters by performing a Principal Coordinate Analysis (PCoA) based on *F*_ST_ estimates in GenAlEx. This multivariate analysis allows the assessment of distances between groups of samples and then represents them in ordination space.

### 2.5 Assignment tests

#### 2.5.1 Inferring origin based on genetic clusters

We used methods previously described to create simulated reference populations representing distinct potential sources of deer and perform genetic assignments of samples to these reference populations (Miller & Walter, 2020). Using simulated reference populations to represent potential sources may reduce bias introduced by uneven sampling of those sources (Karlsson et al., 2014). We created reference populations that represented: (a) captive deer, (b) wild deer, (c) each cluster of wild deer inferred with non‐spatial BCA (*n* = 2), and (d) each cluster of wild deer inferred with spatial BCA (*n* = 8). We created each reference population by simulating 500 individuals using HybridLab (Nielsen et al., 2006), which takes real genotypes as input and creates simulated genotypes by randomly drawing one allele at each locus from each parental population based on their allele frequencies. We used our empiric genotypes of 50 captive deer and 5701 wild deer. We knew which wild deer were assigned to each non‐spatial BCA cluster based on their highest membership‐to‐cluster value (Kierepka & Latch, 2016; Latch et al., 2014). We did not know the spatial BCA cluster assigned to each wild deer because spatial BCA did not run on the full dataset. Therefore, samples not included in spatial BCA were assumed to belong to the genetic cluster inferred for their geographic position. We included captive deer because of the large number of facilities within Pennsylvania (Rorres et al., 2018) and documented differentiation of captive and wild deer in previous analyses (Hernández‐Mendoza et al., 2014; Miller & Walter, 2020).

We confirmed that genetic diversity was similar between simulated reference populations and the source population they represent by calculating descriptive genetic statistics using Geneclass2 v.2.0 (Piry et al., 2004). Statistics examined were the average number of alleles per locus (*N*_A_) and mean Nei's gene diversity (i.e. average expected heterozygosity; Nei, 1977). We tested for deviations from H–W equilibrium assumptions in reference populations using exact tests in Genepop v.4.6 (Raymond & Rousset, 1995) with 1000 Markov chain dememorization steps and *α* = .01. We also assessed the similarity in patterns of genetic diversity between simulated reference populations and real source populations by performing a PCoA based on F_st_ estimates in GenAlEx. When the ordination space showed a simulated reference population to be located near the centroid of the sampling units it was derived from, we considered the reference population to be an adequate representation of the sampling units (Miller & Walter, 2020). The comparisons were: simulated captive population versus captive facilities where samples were collected, simulated wild population versus states where samples were collected, and simulated population for each genetic cluster versus counties within each cluster. We did not include counties or captive facilities from which we obtained fewer than six samples.

We then conducted genetic assignment tests of our samples to simulated reference populations under three scenarios in Geneclass2 v.2.0: *Scenario 1*: captive versus wild, *Scenario 2*: captive versus each cluster inferred with non‐spatial BCA, and *Scenario 3*: captive versus each cluster inferred with spatial BCA. We calculated assignment scores (A) of individuals to reference populations using the partial Bayesian method of Rannala and Mountain (1997), as done by Miller and Walter (2020). We considered the population that produced the highest A as the inferred origin of the individual in question, using a threshold of 0.05 as the minimum score for assignment (this value does not affect calculations, only changes the display of results with assignment scores below 0.05 being shaded in the results table). We repeated this analysis to include a resampling algorithm (Paetkau et al., 2004) simulating 10,000 individuals (default) and using an *α* = .05 (Miller & Walter, 2020), which allowed us to compute assignment probability (*p*) of each individual to each reference population. This *p* allowed us to infer which reference population could be rejected as potential origin, rather than which population constitutes the most likely origin of an individual, estimated previously using A.

#### 2.5.2 Inferring origin based on anthropogenic spatial units

We used the same methods implemented for the three scenarios above to create simulated reference populations based on anthropogenically derived spatial units representing states and physiographic provinces (Fenneman, 1928). We confirmed that genetic diversity was similar between spatial units and simulated reference populations using the previously described methods (PCoA, *N*_A_, mean Nei's gene diversity, and deviations from H‐W equilibrium assumptions). Any anthropogenically derived spatial unit with less than 25 samples was omitted (i.e. Coastal Plain and Interior Low Plateau for physiographic provinces).

## 3 RESULTS

### 3.1 Genetic variability

**FIGURE 2:**
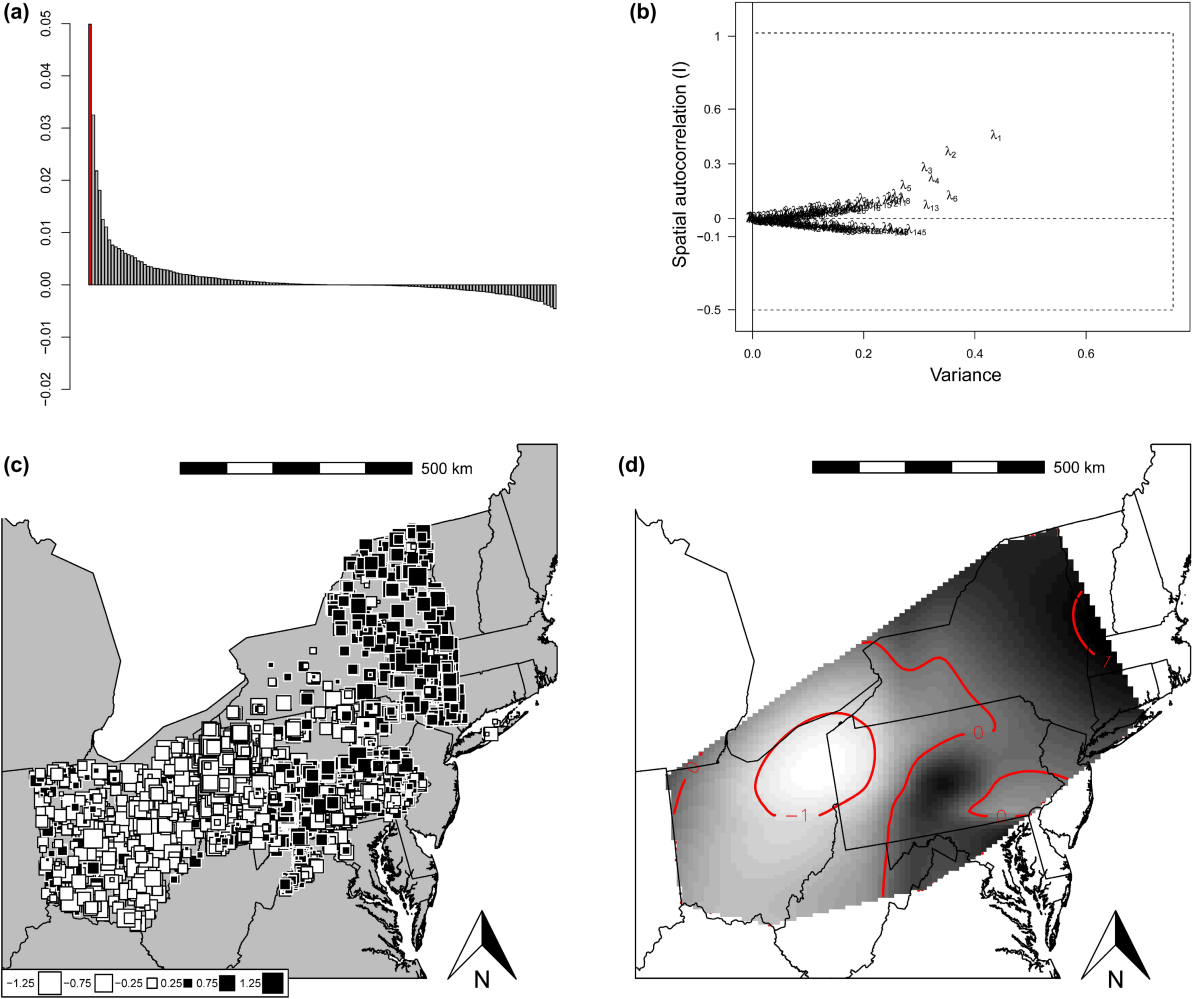
Results of Spatial Principal Component Analysis (sPCA) using Delaunay connectivity network and all samples from wild white‐tailed deer (*Odocoileus virginianus*; *n* = 5701) showing (a) bar plot of eigenvalues with positive ones indicating global structures and negative ones indicating local structures, (b) spatial autocorrelation and variance components of eigenvalues (*λ*) from which we decided to interpret only the first axis, (c) value of each sample along the first sPCA axis, where more positive values are represented by larger black squares and more negative values are represented by larger white squares, and (d) values along the first sPCA axis represented as an interpolation, with darker areas indicating more positive values, and red lines representing isoclines for values of −1, 0 and 1.

We detected global but not local structures with both connectivity networks used in sPCA. When using 999 permutations, we obtained *p*‐value_global_ = .001 and *p*‐value_local_ = 1 for both networks. When using 9999 permutations, we obtained *p*‐value_global_ = .0001 and *p*‐value_local_ = 1 for both networks. Eigenvalue decomposition indicated that only the first principal component should be interpreted for both networks (Figure 2a,b). This axis showed samples with negative values occupying mostly Ohio and western Pennsylvania (white squares, Figure 2c) while samples with positive values were found in eastern New York, central Pennsylvania and Virginia (black squares, Figure 2c), for both connectivity networks. There was considerable overlap between these two groups in Maryland, Virginia, western New York, and eastern Pennsylvania. The pattern did not seem to represent a continuous cline that could create spurious groupings by clustering methods (Figure 2d).

### 3.2 Population structure

#### 3.2.1 Non‐spatial Bayesian clustering algorithm

**FIGURE 3:**
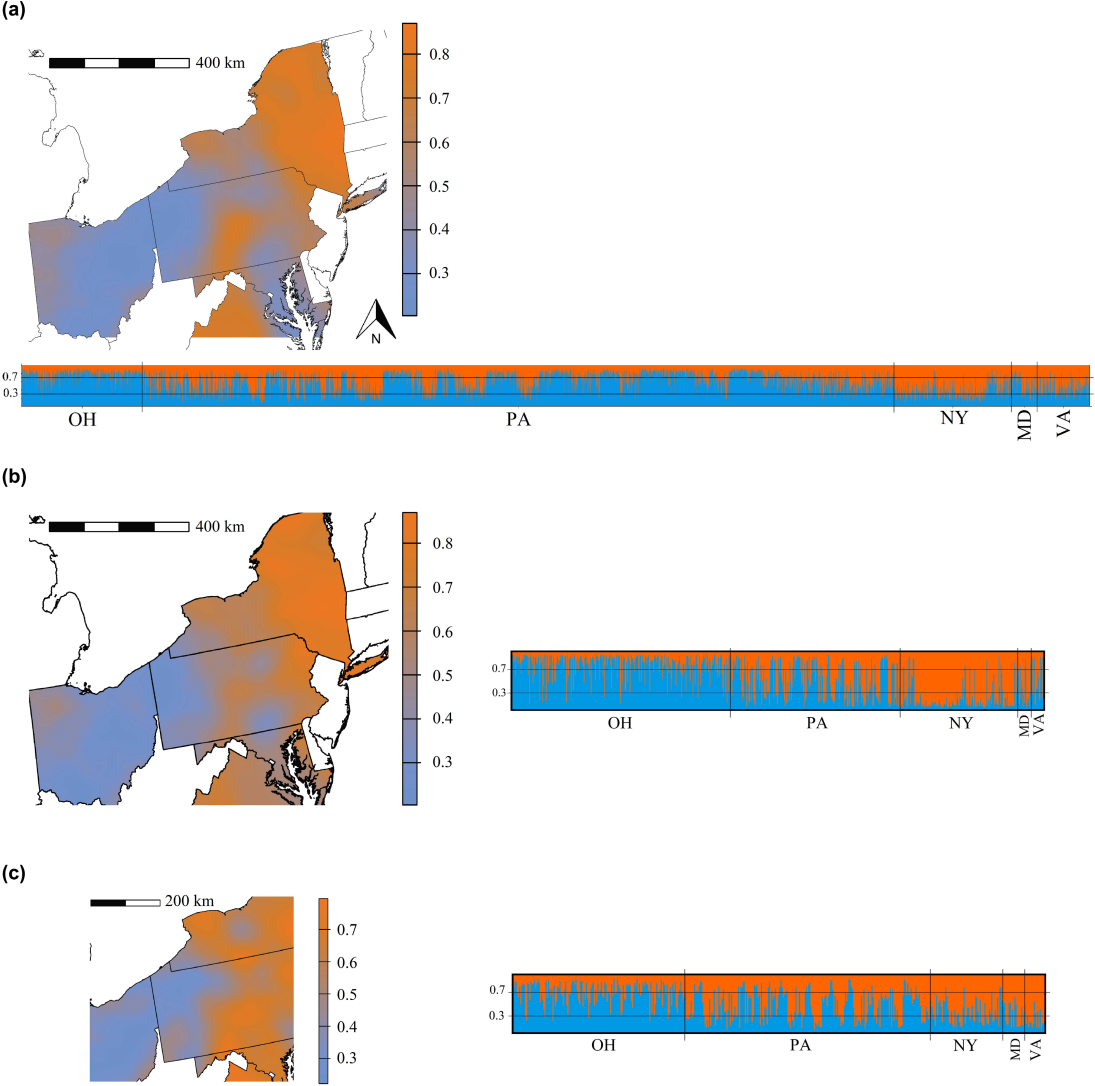
Results of the non‐spatial Bayesian clustering algorithm run in STRUCTURE (Pritchard et al., 2000) for *K* = 2 scenario, for samples of white‐tailed deer (*Odocoileus virginianus*) from the mid‐Atlantic region of the United States. Given that there are only two clusters being represented, membership values (i.e. the percentage of the genome assigned to each cluster) of each individual are inversely proportional between clusters. Membership values are presented as interpolation over the extent of the study area and as a barplot, with each bar representing an individual and colors representing the membership value to a cluster. (a) Membership values obtained using the full dataset of wild white‐tailed deer (*n* = 5701), (b) membership values obtained using our subsample of a maximum of two deer per sex per county (*n* = 750), and (c) membership values obtained using our subsample of a maximum of two deer per sex per county but with samples from extreme locations removed (*n* = 461, representing a 38.5% reduction from the original 750 samples).

There appeared to be 2–3 clusters in the wild deer population, since these consistently had the highest ∆*K* and Pr(*X*|*K*), or the latter only increased slightly at larger Ks, whether we analyzed subsamples (Figure S1a–c) or the full dataset of wild deer (Figure S1d). The subsample of two deer per sex per county showed a peak in Evanno's ∆*K* for *K* = 4 (Figure S1b), but none of these samples had a membership high enough to be assigned to this 4th cluster, supporting *K* = 2 or 3 like all other runs. The STRUCTURE software manual recommends interpreting the smallest *K* in cases where similar values of Pr(*X*|*K*) are obtained for multiple Ks (Pritchard et al., 2010). Therefore, we decided to interpret the structure represented by *K* = 2, which shows a distribution of membership values highly congruent with the interpolation obtained with sPCA (Figures 2d and 3a,b). Under this scenario, one cluster consisted mainly of Ohio and western Pennsylvania, and the other cluster comprised New York, Maryland, Virginia, and Ridge and Valley province in Pennsylvania regardless of subsampling (Figure S2a–d). Regardless of whether we used the subsample of one, two, or five deer per sex per county, 10/10 runs performed for *K* = 2 converged to the same solution.

Removing samples from extreme west and east locations still resulted in inferring genetic structure with a similar geographic pattern (*K* = 2, Figure 3c), which we considered an indication of structure not being an artifact due to IBD. Also for this dataset, 10/10 runs performed for *K* = 2 converged to the same solution. Mantel test of correlation between genetic and geographic distance was significant for this subsample but with very low *R* ^2^ (*p* = .004, *R* ^2^ = .0018).

#### 3.2.2 Spatial Bayesian clustering algorithm

**FIGURE 4:**
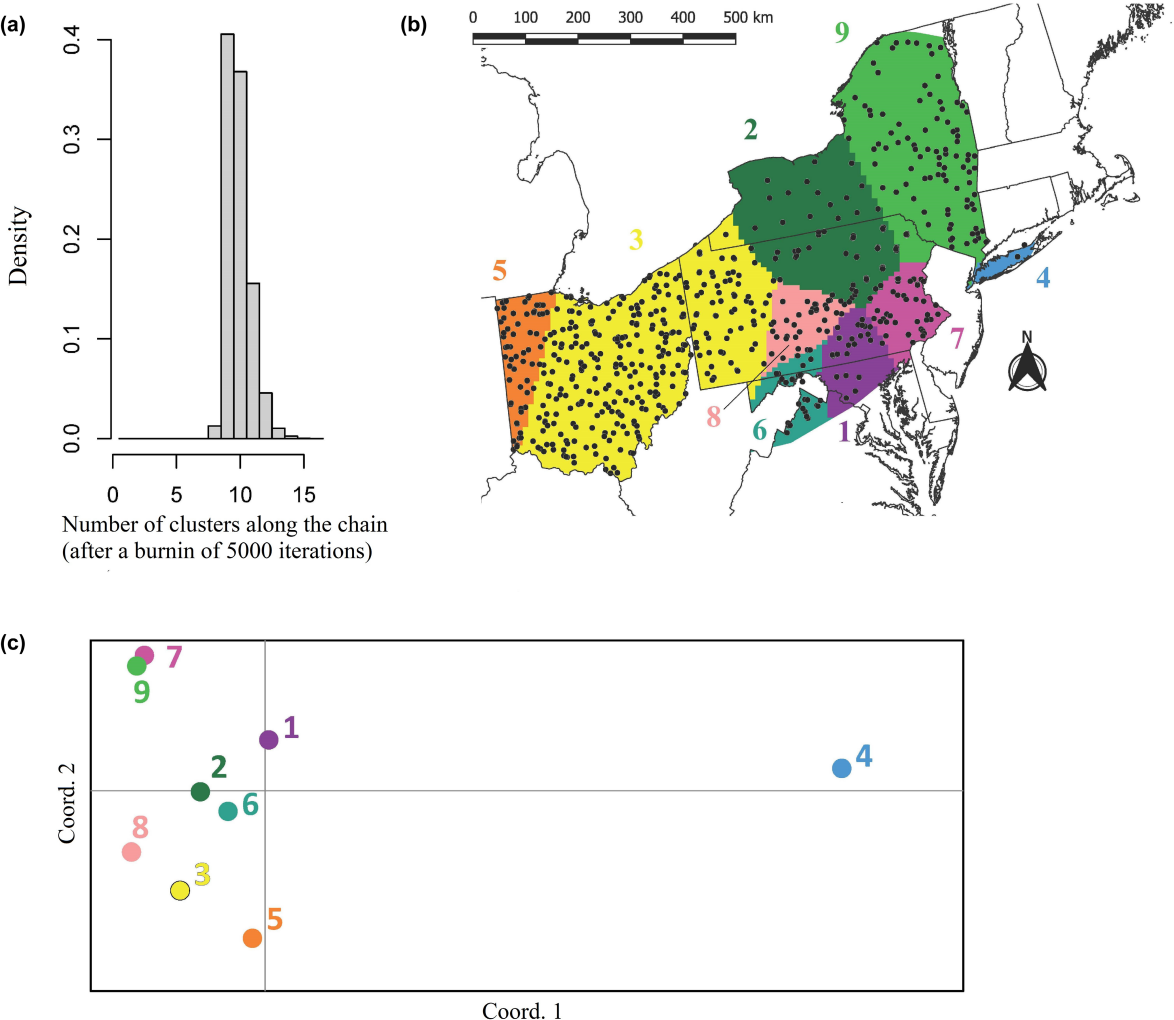
Results of the spatial Bayesian clustering algorithm for the top‐ranked run using our subsample of a maximum of two white‐tailed deer (*Odocoileus virginianus*) per sex per county (*n* = 750): (a) maximum number of populations simulated from the posterior distribution, indicating that *K* = 9 is the solution with highest support; and (b) geographic distribution of the nine inferred clusters shown in different colors and enumerated, with black circles representing sample locations. (c) Results of the Principal Coordinate Analysis showing the nine clusters inferred with spatial Bayesian clustering algorithm in ordination space, with colors matching those used in (b).

**FIGURE 5:**
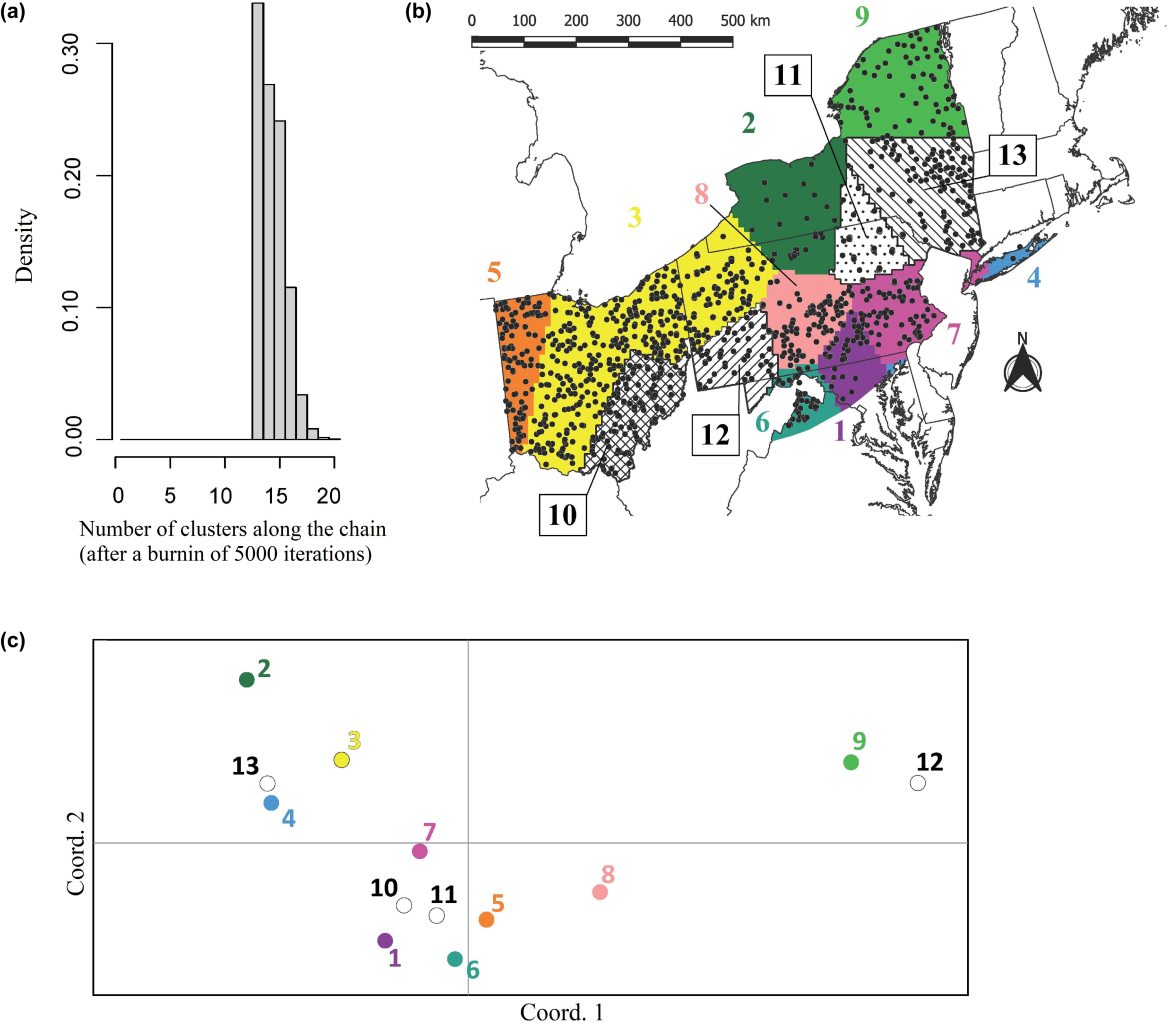
Results of the spatial Bayesian clustering algorithm for the top‐ranked run using our subsample of a maximum of five white‐tailed deer (*Odocoileus virginianus*) per sex per county (*n* = 1557): (a) maximum number of populations simulated from the posterior distribution, indicating that *K* = 13 is the solution with highest support; and (b) geographic distribution of the 13 inferred clusters shown in different colors and enumerated, with black circles representing sample locations. (c) Results of the Principal Coordinate Analysis showing the 13 clusters inferred with spatial Bayesian clustering algorithm in ordination space, with colors matching those used in (b).

When using two deer per sex per county, seven of ten independent runs indicated a maximum *K* of nine, including the top‐ranked run (Figure 4a). One of these clusters contained only six samples, representing Long Island, New York (Figure 4b). Clusters 5, 3, and 8 appeared aligned in the PCoA ordination space, a pattern congruent with their west‐to‐east geographic distribution (Figure 4c). Results from PCoA also showed cluster 4 (Long Island) as the most divergent, while clusters 7 and 9 (geographically adjacent and representing the eastern extent of our samples) were adjacent in the ordination space (Figure 4c). When using five deer per sex per county, the divisions between these nine clusters were mostly maintained, but three clusters were further partitioned, with the top‐ranked run indicating a maximum *K* of 13 (Figure 5a,b). However, the distribution of these clusters in the PCoA ordination space was incongruent with their geographic distribution (Figure 5c). For example, clusters 10 and 11 were located next to each other in the ordination space even though they belong to distant geographic areas, while the opposite can be observed for clusters 9 and 13. Therefore, we decided to follow a conservative approach and interpret the scenario with fewer clusters (*K* = 9), taking into account that both subsets of samples used in the analysis recovered roughly the same geographic extension for each of these clusters.

### 3.3 Assignment tests

#### 3.3.1 Inferring origin based on genetic clusters

Simulated populations had values of Nei's gene diversity and N_A_ comparable to the empirical groups they represent (Table 1, maximum difference in *N*_A_ = 1.1 alleles, maximum difference in Nei's gene diversity = 0.007). We did not create a reference population for one of the clusters inferred with spatial BCA (Cluster 4, Long Island, Figure 4b) because it was composed of only 17 samples. The simulated population representing cluster 3 inferred with spatial BCA showed one locus departing from H–W equilibrium assumptions (OarFCB193, *p*‐value = .0052). Results from PCoA showed that simulated populations occupied a position near the centroid of the locations they represent (Figures S3 and S4).

**TABLE 1:** Comparison between each simulated reference population and the empiric group from which it derives, for samples of white‐tailed deer (*Odocoileus virginianus*) from the mid‐Atlantic region of the United States.

	*N*	*N*_A_	*D*
Captive	500/50	10.30/10.30	0.807/0.812
Wild	500/5701	14.30/15.20	0.852/0.850
Cluster A non‐spatial BCA	500/2825	14.20/15.10	0.847/0.849
Cluster B non‐spatial BCA	500/2876	13.60/14.70	0.840/0.839
Cluster 1 spatial BCA	500/480	13.40/13.80	0.842/0.841
Cluster 2 spatial BCA	500/848	13.90/14.10	0.849/0.851
Cluster 3 spatial BCA	500/1626	13.90/14.30	0.831/0.831
Cluster 5 spatial BCA	500/124	12.40/12.40	0.825/0.832
Cluster 6 spatial BCA	500/495	13.90/13.90	0.838/0.840
Cluster 7 spatial BCA	500/362	12.80/13.20	0.834/0.836
Cluster 8 spatial BCA	500/1202	13.90/14.10	0.838/0.839
Cluster 9 spatial BCA	500/547	13.30/13.40	0.844/0.841

*Note*: *N*, number of individuals (simulated/empiric); *N*_A_, mean number of alleles (for simulated individuals/for empiric individuals); *D*, mean Nei's genetic diversity (for simulated individuals/for empiric individuals). We did not create a simulated population for Cluster 4 (inferred using spatial Bayesian clustering algorithm (BCA) in GeneLand) because it was represented by only 17 samples.

Dividing individuals into either captive or wild simulated populations (Scenario 1) had an overall success rate of 93.5% (Table 2). Correctly assigned captive deer had high assignment scores to the captive population (average *A* = 95.0) while those incorrectly assigned to the wild population had an average *A* of 74.3. Wild deer showed the same pattern: correctly assigned deer had high assignment scores (average *A* = 97.2) while incorrectly assigned ones had average *A* of 78.6. Also in Scenario 1, 7.2% of wild deer had *p* < .05 to the wild reference population, therefore incorrectly rejecting it as their source (Table 2). In the case of captive deer, 12% had *p* < .05 to the captive reference population, erroneously rejecting it as their source.

**TABLE 2:** Genetic assignment tests to simulated reference populations based on genetic clusters for samples of white‐tailed deer (*Odocoileus virginianus*) from the mid‐Atlantic region of the United States.

Origin of samples	% of correct assignments based on *A*	Average *A* to correct source	Average *A* to incorrect source	% of assignments rejecting correct source based on *p*
*Scenario 1*	93.5			
Captive	96.0	95.0	74.3	12.0
Wild	93.5	97.2	78.6	7.2
*Scenario 2*	90.0			
Captive	96.0	91.0	81.1	12.0
Wild Cluster A from non‐spatial BCA	92.5	89.8	63.2	8.2
Wild Cluster B from non‐spatial BCA	87.4	87.2	70.1	6.8
*Scenario 3*	61.8			
Captive	90.0	83.6	68.9	14.0
Wild Cluster 1 from spatial BCA	65.2	81.2	59.2	8.1
Wild Cluster 2 from spatial BCA	50.7	69.5	58.9	7.9
Wild Cluster 3 from spatial BCA	57.9	68.1	58.0	5.5
Wild Cluster 5 from spatial BCA	75.8	81.6	55.4	8.9
Wild Cluster 6 from spatial BCA	67.7	81.0	61.7	6.9
Wild Cluster 7 from spatial BCA	57.5	72.4	56.7	7.5
Wild Cluster 8 from spatial BCA	60.6	77.3	58.0	6.0
Wild Cluster 9 from spatial BCA	82.1	83.9	56.2	6.0

*Note*: *A*, assignment score calculated using the Rannala and Mountain (1997) method; *p*, assignment probability calculated using the Paetkau et al. (2004) method.

We also obtained a high percentage of correct assignments in Scenario 2, with an overall success rate of 90.0%, while the percentage of correct assignments to reference populations ranged between 87.4 and 96.0. Tests were much less efficient when assigning wild deer into reference populations in Scenario 3 (percentage of correct assignments ranged between 50.7 and 82.1), although the percentage of correct assignments for captive deer remained high (90.0%).

#### 3.3.2 Inferring origin based on anthropogenic spatial units

For each simulated reference population using anthropogenically derived spatial units, Nei's gene diversity and N_A_ were comparable to values for the sampling units they were based on (Table 3). The simulated population representing the Adirondack province had one locus departing from *H*–*W* equilibrium assumptions (BM6438, *p*‐value = .0003), while all other reference populations and loci met these assumptions. Reference populations were approximately centroidal when using PCoA by state and physiographic province, except for Blue Ridge (Figures S5 and S6).

**TABLE 3:** Comparison between each simulated reference population and the empiric anthropogenic spatial units from which it derives (state or physiographic province) for samples of white‐tailed deer (*Odocoileus virginianus*) from the mid‐Atlantic region of the United States.

	*N*	*N*_A_	*D*
*State*			
Pennsylvania	500/4013	14.10/14.80	0.848/0.849
Maryland	500/138	13.70/13.70	0.845/0.845
Ohio	500/643	13.40/13.90	0.830/0.831
Virginia	500/281	13.80/13.80	0.836/0.834
New York	500/626	13.70/13.90	0.843/0.844
*Physiographic Province*			
Adirondack	500/89	11.30/11.30	0.835/0.843
Appalachian Plateaus	500/2484	14.00/14.50	0.843/0.843
Blue Ridge	500/62	11.40/11.40	0.821/0.826
Central Lowland	500/374	13.20/13.60	0.832/0.835
New England	500/40	10.70/10.70	0.819/0.829
Piedmont	500/491	13.10/13.40	0.845/0.844
St. Lawrence	500/58	10.80/10.80	0.828/0.835
Valley and Ridge	500/2082	14.60/14.90	0.846/0.847

*Note*: *N*, number of individuals (simulated/empiric); *N*_A_: mean number of alleles (for simulated individuals/for empiric individuals); *D*, mean Nei's genetic diversity (for simulated individuals/for empiric individuals).

The overall percentage of correct assignments was 53.0 when simulated reference populations represented captive and five states, and 53.8 when simulated reference populations represented captive and the eight physiographic provinces (Table 4). The percentage of correct assignments for captive deer was the same when wild reference populations represented states or physiographic provinces (86%). As for wild deer, the percentage of correct assignments to states ranged between 43.8 and 80.7, while percentage of correct assignments to physiographic provinces ranged between 44.3 and 90.0 (the latter representing assignments to New England province, in the far northeast of our study area). Also for wild deer, the percentage of assignments rejecting correct population of origin based on *p* was slightly higher when dividing the wild population into physiographic provinces.

**TABLE 4:** Genetic assignments to simulated reference populations based on anthropogenically‐derived spatial units for samples of white‐tailed deer (*Odocoileus virginianus*) from the mid‐Atlantic region of the United States.

Origin of samples	% of correct assignments based on *A*	Average *A* to correct population	Average *A* to incorrect populations	% of assignments rejecting correct population of origin based on *p*
*States*	53.0			
Captive	86.0	89.6	57.5	12.0
Pennsylvania	43.8	66.8	67.3	7.0
Maryland	57.2	79.3	57.2	7.2
Ohio	70.9	77.2	60.5	6.4
Virginia	74.0	84.9	60.7	5.7
New York	80.7	90.1	60.5	7.2
*Physiographic Provinces*	53.8			
Captive	86.0	87.5	70.9	12.0
Adirondack	75.3	79.7	65.8	6.7
Appalachian Plateaus	44.3	65.9	61.8	6.6
Blue Ridge	88.7	91.9	50.7	11.3
Central Lowland	59.9	72.5	58.7	5.9
New England	90.0	87.2	67.1	7.5
Piedmont	58.9	75.7	63.7	7.3
St. Lawrence	82.8	82.9	67.4	8.6
Valley and Ridge	58.5	80.1	61.8	7.4

*Note*: *A*, assignment score calculated using the Rannala and Mountain (1997) method; *p*, assignment probability calculated using the Paetkau et al. (2004) method.

## 4 DISCUSSION

Inferring boundaries of demes can provide useful information for disease control, for example by delimiting the extent of the area most likely at risk of disease spread during an outbreak. However, the assessment of genetic structure in white‐tailed deer can be obscured by human activities that have altered the genetic composition of natural populations, such as deer farming combined with the escape of captive deer (Miller & Walter, 2020). Numerous historical translocations are also likely to have left a mark on the genetic composition of wild deer populations (Chafin et al., 2021; Leberg et al., 1994), generating confounding effects when inferring contemporary gene flow and its landscape barriers. We found evidence of population structure using both non‐spatial and spatial BCA. It is not unexpected to infer more clusters using a spatial BCA; however, both methods can complement each other and allow the inference of different levels of hierarchical genetic structure (Guillot, Mortier, & Estoup, 2005).

Population structure as determined by genetic variability across the northeastern United States supports previous findings that there is low genetic differentiation while also suggesting that some population structure exists (Bauder et al., 2021; Miller, Miller‐Butterworth, et al., 2020). Generally, low genetic differentiation would suggest that movement of disease across the landscape would be expected in this highly mobile large mammal. The pattern of genetic structure we found by applying a non‐spatial Bayesian clustering algorithm was highly congruent with the pattern of genetic variability obtained with sPCA. We obtained the same pattern of genetic structure using the full dataset and three different subsamples, therefore concluding that these subsamples are representative of the underlying structure. The clearest divide between clusters in the *K* = 2 scenario was between Ohio‐western Pennsylvania vs central valleys of Pennsylvania clustering with all other states. The Allegheny mountains were a potential barrier between clusters, especially in the northern region. Another factor that could be contributing to the separation between the west and east portions of Pennsylvania is the Allegheny River, which has been shown to act as a semipermeable barrier for white‐tailed deer in the past (Long et al., 2010). This is also around the division between the Appalachian Plateaus and Valley and Ridge physiographic provinces. Restocking of deer in Ohio from Pennsylvania captive cervid facilities and vice versa occurred in the early 1900s, which could at least partially explain the general similarity between Ohio and west Pennsylvania populations (Chapman, 1938; Pennsylvania Game Commission, 1995).

Using a spatial BCA we identified a maximum of nine genetic clusters with similar geographic extent regardless of inclusion of two or five deer per sex per county. For the latter subset, four additional clusters were retrieved but their genetic distances summarized by PCoA were inconsistent with their geographic distances. These results could indicate an effect of sampling bias over the clustering algorithm: including five deer per sex per county resulted in some counties being represented by 10 samples, while others only have one sample. For the nine clusters that were consistent in both subsets of samples, the pattern retrieved was congruent with known barriers to white‐tailed deer gene flow. Samples from Long Island (cluster 4) were grouped separately from other samples collected geographically close, which is consistent with the presence of the natural barrier between the island and the mainland. The division between the two clusters present in Ohio (clusters 3 and 5) appears to match the Interstate 75 highway, a result consistent with a previous study including samples exclusively from Ohio (Bauder et al., 2021). Another major highway, Interstate 81, could also account for the separation between clusters 2 from 9, 2 from 7, and 8 from 1 (Figure 4), while interstate 99 could explain the separation between clusters 3 and 8. These apparent barriers to gene flow can be incorporated into CWD control practices, noting that white‐tailed deer management units do not exactly match the population structure we found (Karns et al., 2015; New York State Department of Environmental Conservation, 2021; Pennsylvania Game Commission, 2023) likely due to multi‐species management units, public compliance, and other considerations. Our results identified nine Genetic Management Units (GMUs) that could be considered by state management agencies for more effective deer population management in the presence of CWD. For example, the recent discovery of CWD in captive and wild deer in GMU 3 in Ohio would suggest that the risk of CWD expanding into eastern Ohio would be more likely than expanding to western Ohio (GMU 5; Figure 5) or the state of Indiana due to the potential limited gene flow between the two GMUs due to Highway 75. Because CWD has been documented to decrease populations for some species (Edmunds et al., 2016), management of deer populations cannot exist in a vacuum without the influence of disease impacting populations. To our knowledge, states experiencing CWD have not altered management units statewide but have added disease management units to existing infrastructure for more detailed monitoring. The development of GMUs crossing state boundaries may also lead to fostering cooperation between multiple state agencies (Davies et al., 2021).

Miller and Walter (2020) obtained high success classifying samples into captive compared to wild origin using genetic assignments with the same captive deer samples we used but with a smaller fraction of wild deer samples. Because our samples of wild deer represented a much larger area, and therefore, more genetic variability, we did not know if our assignment tests would be as effective as the ones conducted by these authors. We obtained a high rate of correct classifications into captive or wild origin using a method based on assignment scores, but the method using assignment probabilities performed poorly: the correct population of origin was rejected for a high percentage of samples in all scenarios.

The efficiency of assignments to clusters inferred via non‐spatial BCA was higher than the efficiency of assignments to anthropogenically derived spatial units, but the large geographic extent of both clusters means these assignments are not very informative to infer the geographic origin of deer, making the method unlikely to become a useful tool for CWD management. The overall percentage of correct assignments to clusters inferred via spatial BCA was also higher than for anthropogenically derived spatial units (61.8 vs. ∼53, respectively). Assignments to each of these genetic clusters, however, did not have a high percentage of success. This indicates that differentiation among clusters, although high enough to be detected by spatial BCA, could be insufficient to confidently assign individuals to clusters using our methodology. It is possible that about 62% accuracy in assignment is better than not having any assignment to the origin for management agencies when a potential new CWD‐positive deer is discovered.

### 4.1 Caveats and future directions

Although low accuracy (50.7%–82.1%) occurred when assigning deer from our entire dataset to spatial BCA clusters, it is important to note that we assumed the correct population of origin for many of these samples based on their geographic position inside clusters inferred with subsets of samples. Additional efforts to increase the processing of more samples per defined spatial area with cyber‐infrastructure may address some of the drawbacks in our study. In addition, low accuracy with more divisions may prevent splitting populations into too many groups, especially without a genetic basis. An additional caveat is the low number of captive facilities for our comparison of captive to wild samples could have biased results, however, this likely was mitigated because of the use of simulated reference populations (Miller & Walter, 2020).

## 5 CONCLUSIONS

An understanding of the population structure of white‐tailed deer is necessary in the context of disease transmission and spread throughout a region. Widespread gene flow among white‐tailed deer across the northeastern United States documented in our research may not prevent disease transmission across the region, but some population structure is present. Developing GMUs based on our estimated genetic clusters will likely be useful for long‐term monitoring of CWD across this region in the future. For example, our results can be used to identify areas of likely CWD spread in the presence of an outbreak and identify areas at lower risk due to their isolation. Future research to expand upon these results may include examining gene flow among these GMUs and determining potential landscape features that may act as barriers in the region. Evaluation of samples with an extensive panel of single‐nucleotide polymorphisms (SNPs) could improve assignment accuracy to the inferred clusters, representing contemporary gene flow and its landscape barriers. Due to the cost and amount of DNA required for SNPs, an initial assessment with microsatellites could provide information on clustering of deer populations across a region, which could inform the sample locations selected for future SNP assessment in a region.

## AUTHOR CONTRIBUTIONS

**W. David Walter:** Conceptualization (lead); data curation (lead); formal analysis (equal); funding acquisition (lead); investigation (lead); methodology (equal); project administration (lead). **Alberto Fameli:** Conceptualization (supporting); data curation (supporting); formal analysis (equal); funding acquisition (supporting); investigation (supporting); methodology (equal); project administration (supporting). **Kelly Russo‐Petrick:** Conceptualization (supporting); data curation (supporting); formal analysis (supporting); funding acquisition (supporting); investigation (supporting); methodology (supporting); project administration (supporting). **Jessie E. Edson:** Conceptualization (supporting); data curation (supporting); formal analysis (supporting); funding acquisition (supporting); investigation (supporting); methodology (supporting); project administration (supporting). **Christopher S. Rosenberry:** Conceptualization (supporting); data curation (supporting); formal analysis (supporting); funding acquisition (supporting); investigation (supporting); methodology (supporting); project administration (supporting). **Krysten L. Schuler:** Conceptualization (supporting); data curation (supporting); formal analysis (supporting); funding acquisition (supporting); investigation (supporting); methodology (supporting); project administration (supporting). **Michael J. Tonkovich:** Conceptualization (supporting); data curation (supporting); formal analysis (supporting); funding acquisition (supporting); investigation (supporting); methodology (supporting); project administration (supporting).

## CONFLICT OF INTEREST STATEMENT

The authors declare no conflicts of interest.

## Supporting information


Figure S1

Figure S2

Figure S3

Figure S4

Figure S5

Figure S6

Appendix S1

## Data Availability

All code used to process the data is archived at https://code.usgs.gov/cooperativeresearchunits/wtd‐population‐genetic‐structure; Walter et al. (2023). Data are permanently archived at https://www.sciencebase.gov/catalog/item/653179e0d34ee4b6e05bad29.
