# Supplementary figures and images for: Large‐scale assessment of genetic structure to assess risk of populations of a large herbivore to disease
Source: Ecol Evol. 2024 May 20;14(5):e11347. doi: 10.1002/ece3.11347 (PMC11106048; doi:10.1002/ece3.11347)

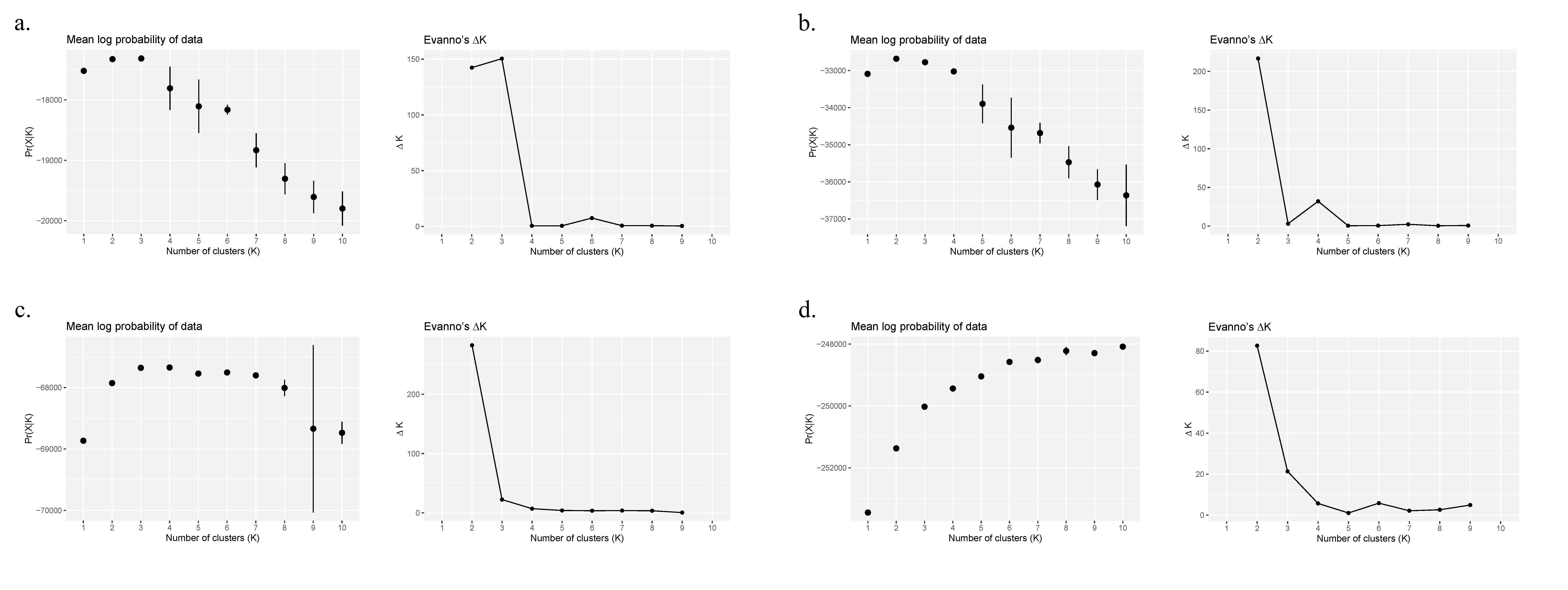

Supplement: Supplementary file 1 — Figure S1 [file ECE3-14-e11347-s007.jpg]

a.

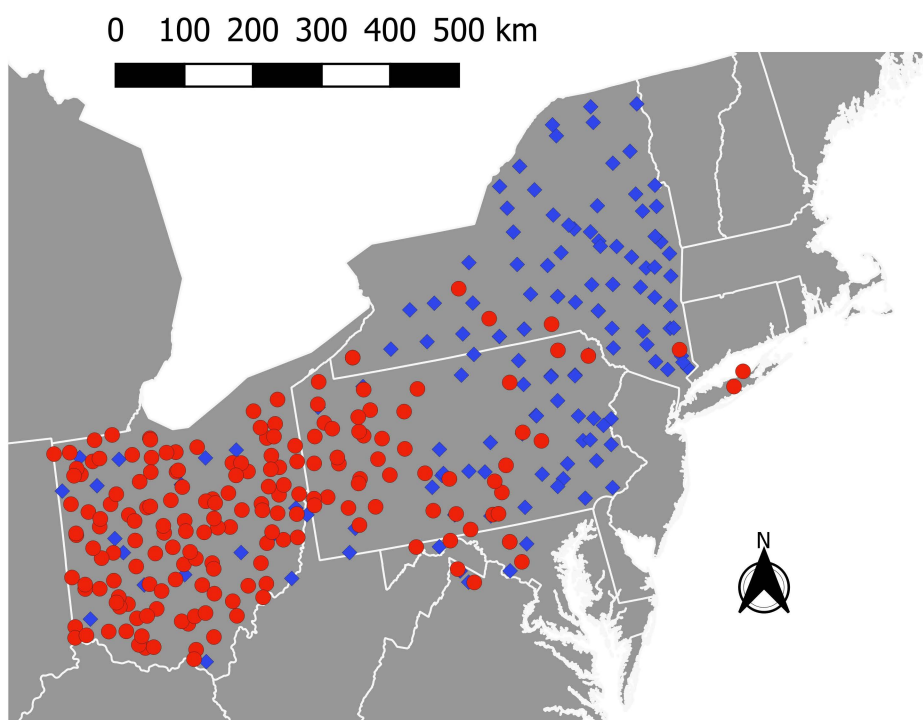

b.

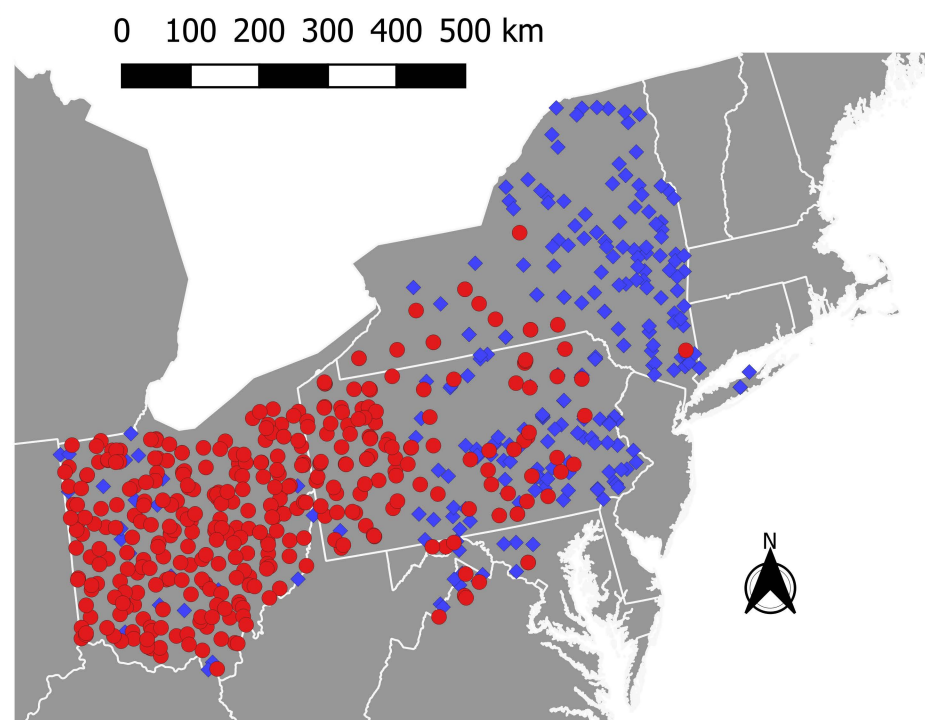

c.

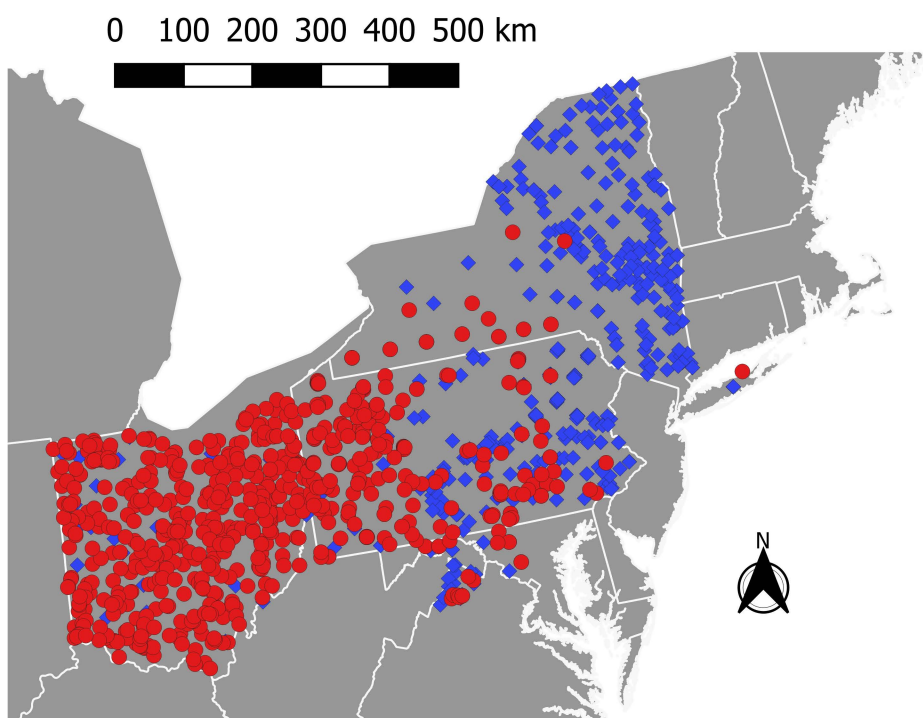

d.

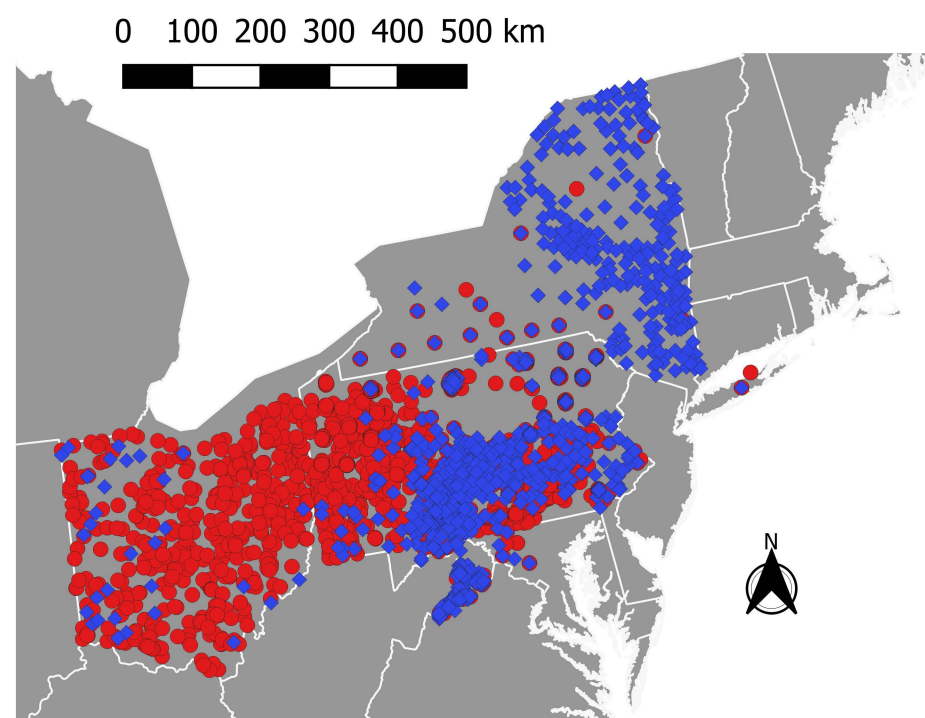

Supplement: Supplementary file 2 — Figure S2 [file ECE3-14-e11347-s001.pdf]

a.

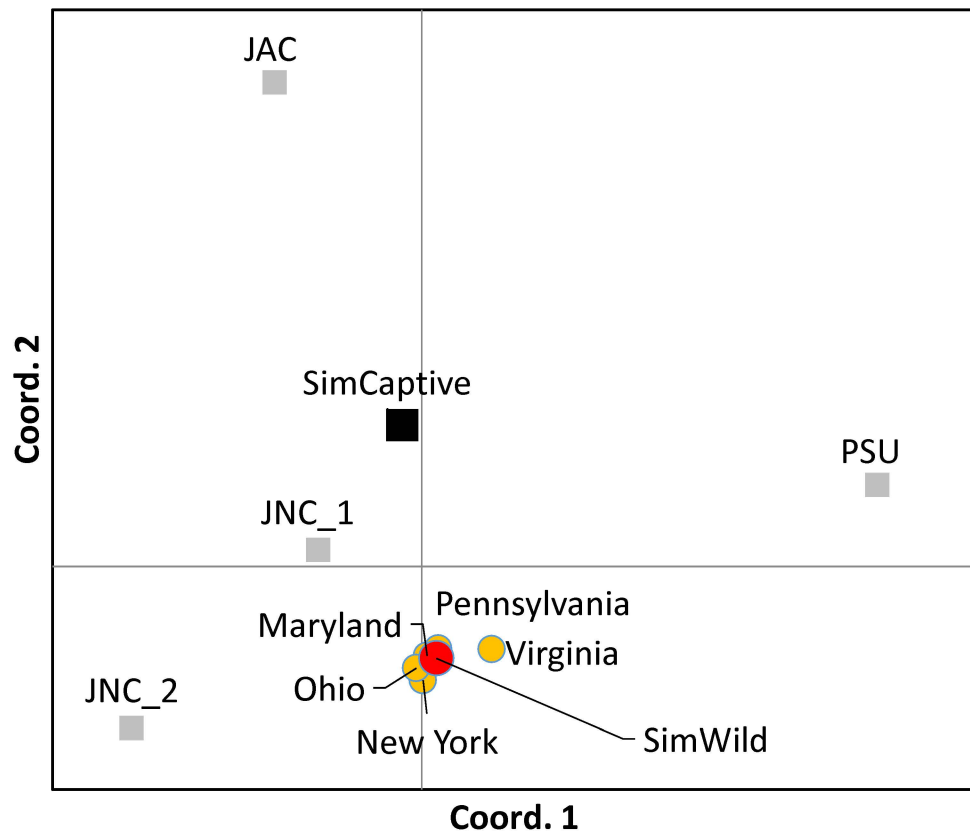

b.

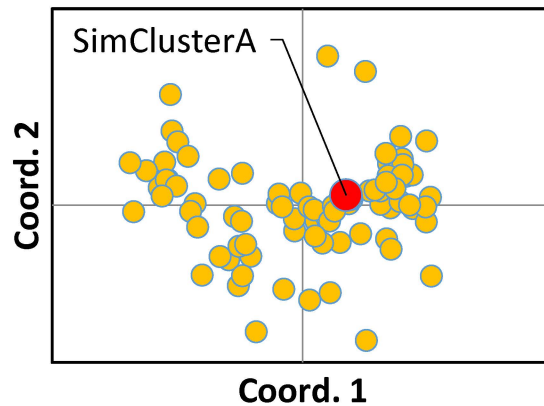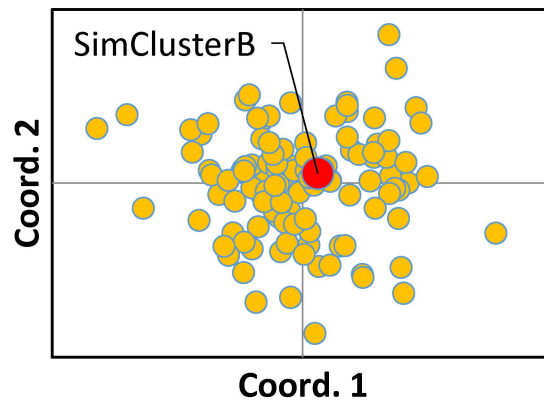

Supplement: Supplementary file 3 — Figure S3 [file ECE3-14-e11347-s005.pdf]

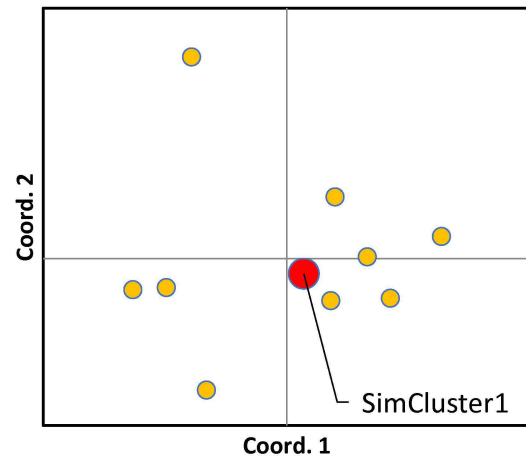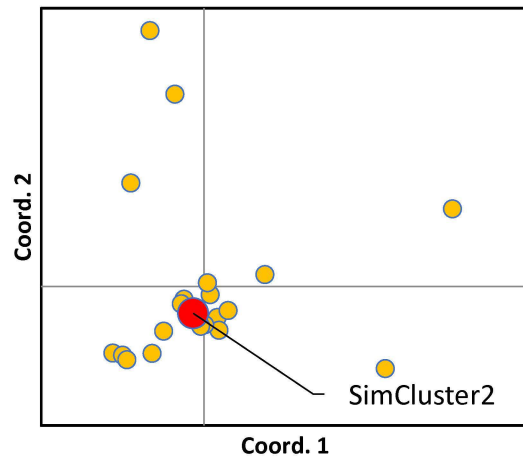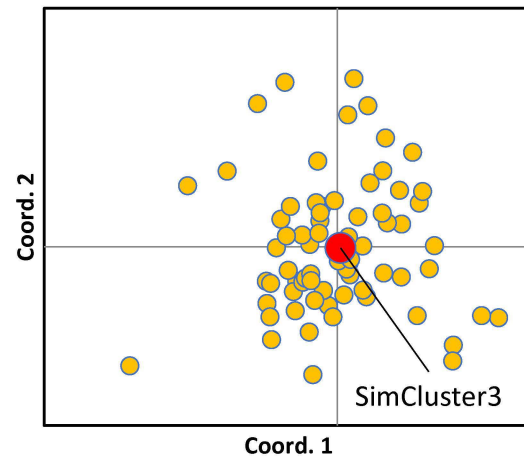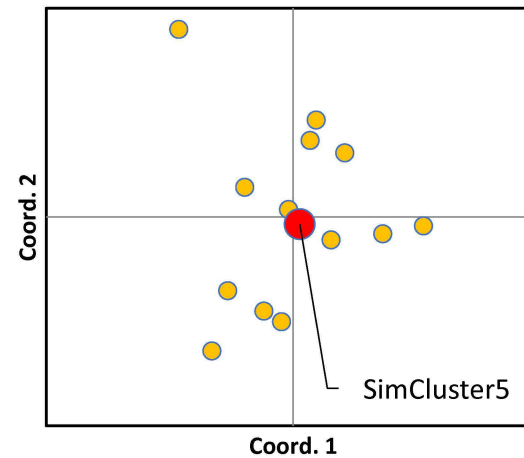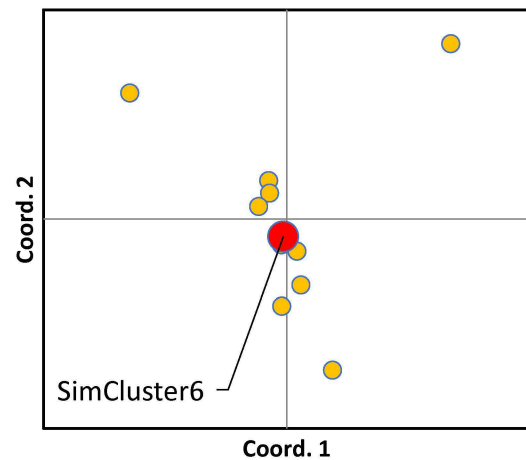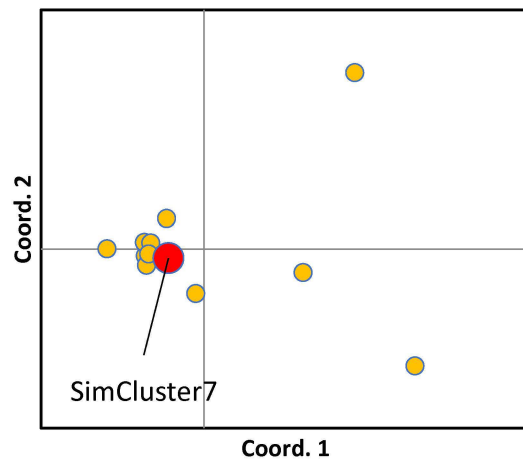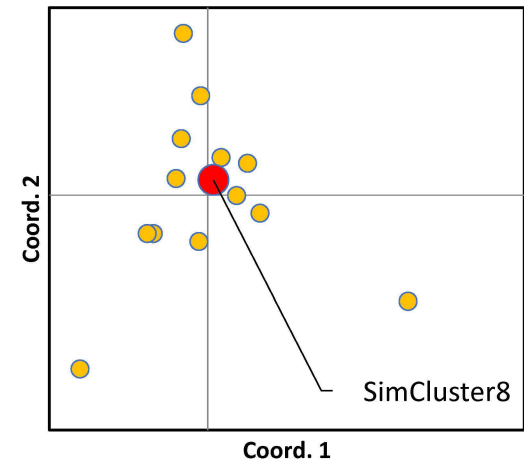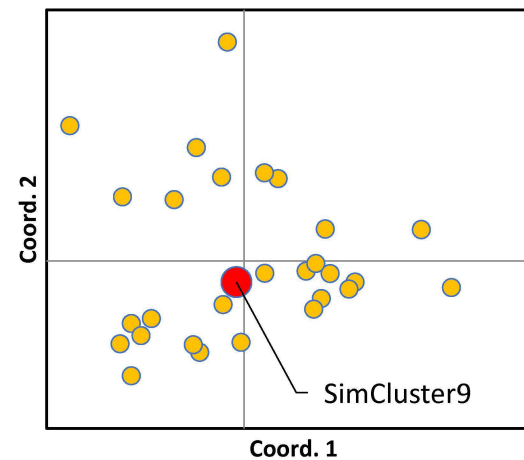

Supplement: Supplementary file 4 — Figure S4 [file ECE3-14-e11347-s006.pdf]

Pennsylvania

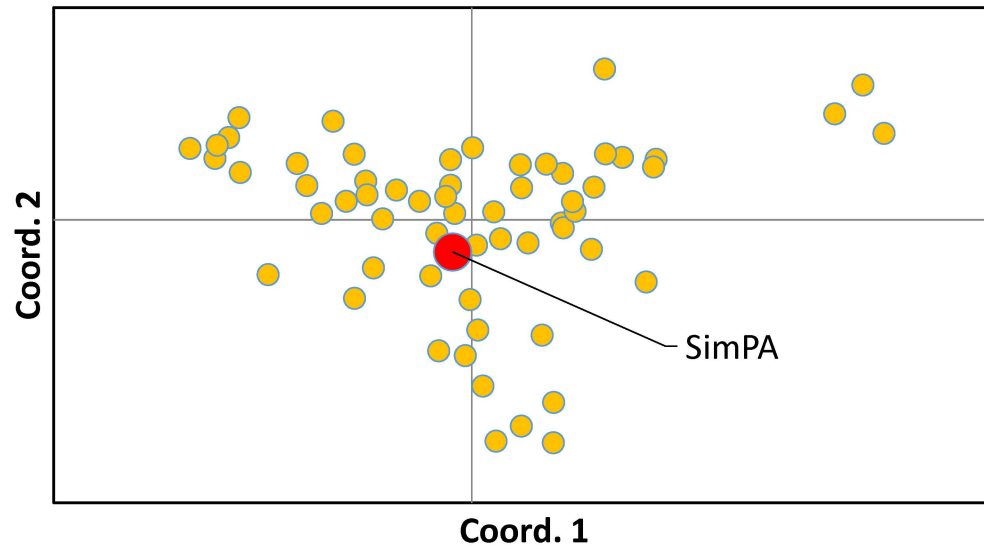

Ohio

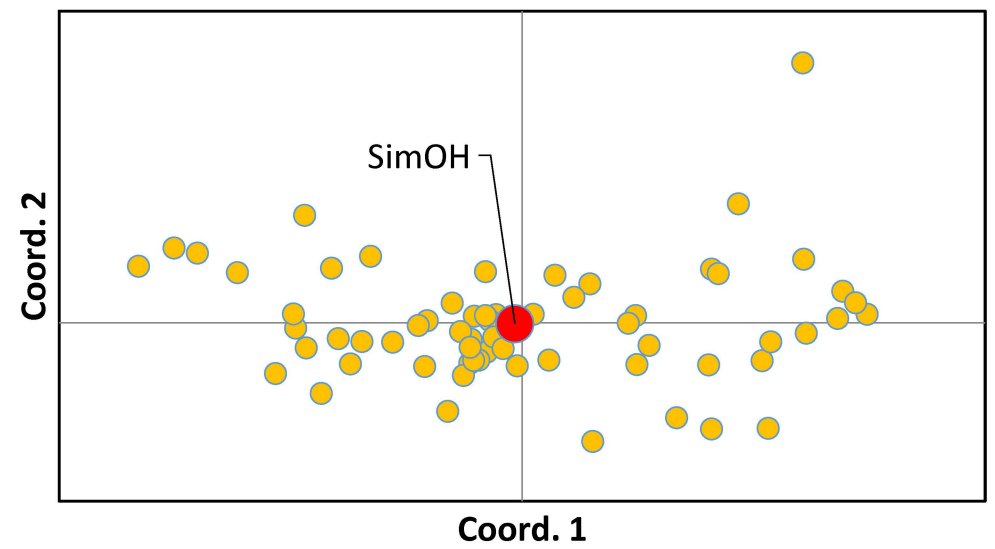

New York

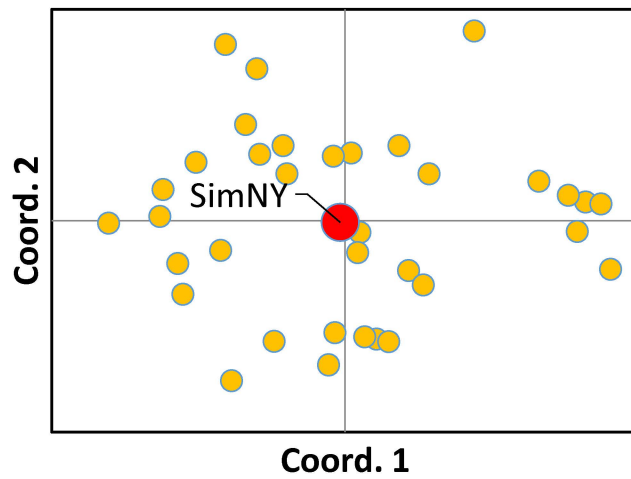

Maryland

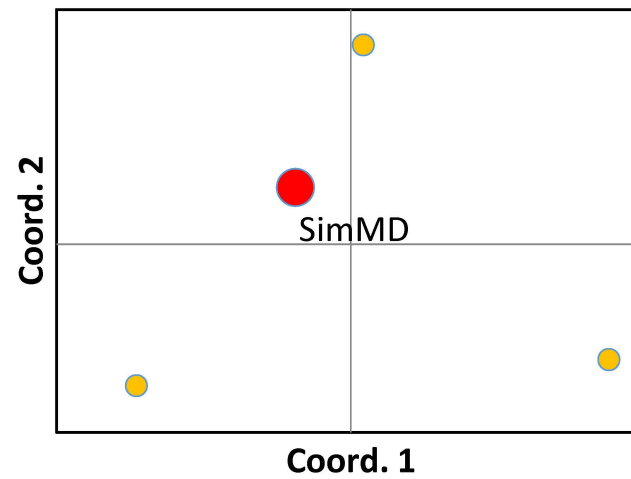

Virginia

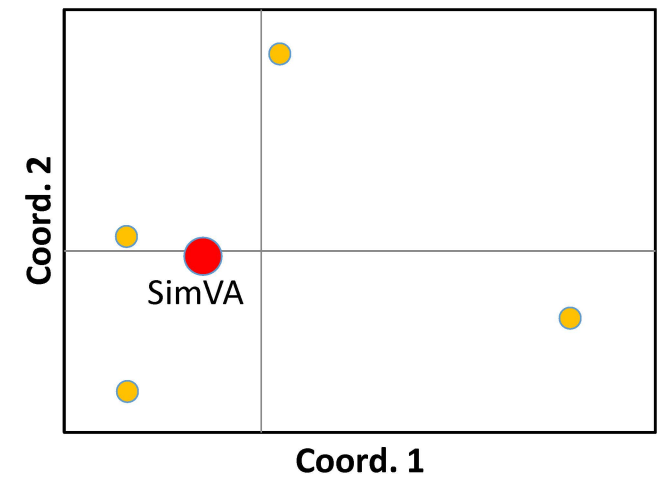

Supplement: Supplementary file 5 — Figure S5 [file ECE3-14-e11347-s002.pdf]
