# Supplementary material for: Large‐scale assessment of genetic structure to assess risk of populations of a large herbivore to disease
Source: Ecol Evol. 2024 May 20;14(5):e11347. doi: 10.1002/ece3.11347 (PMC11106048; doi:10.1002/ece3.11347)
Supplement: Supplementary file 6 — Figure S6 [file ECE3-14-e11347-s003.pdf]

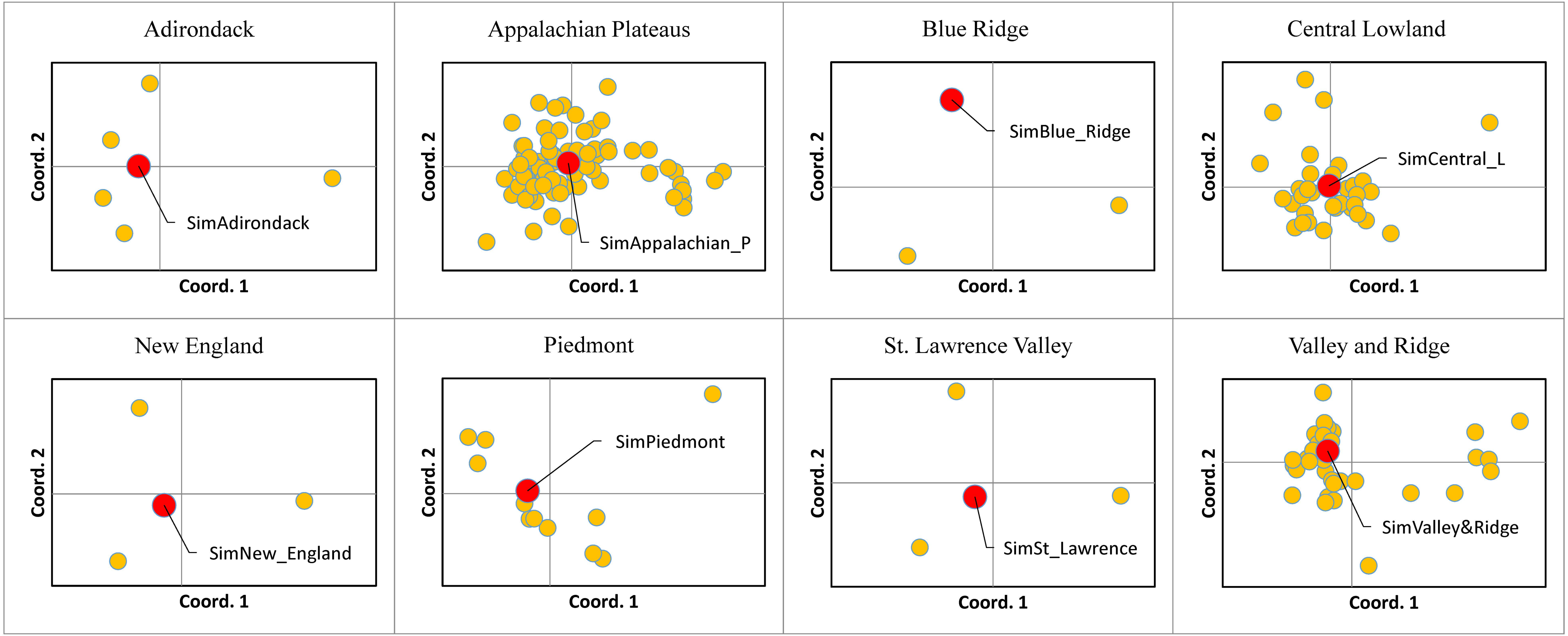

Adirondack
SimAdirondack
Appalachian Plateaus
SimAppalachian_P
Blue Ridge
SimBlue_Ridge
Central Lowland
SimCentral_L
New England
SimNew_England
Piedmont
SimPiedmont
St. Lawrence Valley
SimSt_Lawrence
Valley and Ridge
SimValley&Ridge
Coord. 1
Coord. 2
