# Supplementary material for: Large‐scale assessment of genetic structure to assess risk of populations of a large herbivore to disease
Source: Ecol Evol. 2024 May 20;14(5):e11347. doi: 10.1002/ece3.11347 (PMC11106048; doi:10.1002/ece3.11347)
Supplement: Supplementary file 7 — Appendix S1 [file ECE3-14-e11347-s004.docx]

**Supplementary Information**

**Large-scale assessment of genetic structure to assess risk of populations of a large herbivore to disease**

W. David Walter, Alberto Fameli, Kelly Russo-Petrick, Jessie E. Edson, Christopher S. Rosenberry, Krysten L. Schuler, Michael J. Tonkovich

**Supplementary Figure S1.** Results of the non-spatial Bayesian clustering algorithm run in STRUCTURE (Pritchard et al. 2000) used to determine the most likely number of genetic clusters (K) for samples of white-tailed deer (*Odocoileus virginianus*) collected from the mid-Atlantic region of the United States from 2014 to 2022. Plots show mean of estimated Ln probability for data and Evanno’s ∆K for (a) one deer per sex per county (n=399), (b) two deer per sex per county (n=750), (c) five deer per sex per county (n=1,557), and (d) full dataset (n=5,701)

**Supplementary Figure S2.** Results of the non-spatial Bayesian clustering algorithm run in STRUCTURE (Pritchard et al. 2000) for K=2 scenario for samples of white-tailed deer (*Odocoileus virginianus*) from the mid-Atlantic region of the United States. Individuals assigned to one of the clusters are represented with red circles, while individuals assigned to the other cluster are represented with blue diamonds. Figures are as follows: (a) one deer per sex per county (n=399), (b) two deer per sex per county (n=750), (c) five deer per sex per county (n=1,557), and (d) full dataset (n=5,701). Individuals that did not have membership of ≥0.7 were not mapped.

**Supplementary Figure S3.** Principal Coordinate Analysis comparing simulated reference populations and sampling locations represented for samples of white-tailed deer (*Odocoileus virginianus*) from the mid-Atlantic region of the United States. (a) Simulated population of captive deer (SimCaptive, black square) with the captive facilities represented (gray squares) and simulated population of wild deer (SimWild, red circle) with states represented (orange circles). (b) Simulated populations of wild deer (red circles) for each cluster inferred with non-spatial Bayesian clustering algorithm (top: Cluster A; bottom: Cluster B) with counties represented (orange circles).

**Supplementary Figure S4.** Principal Coordinate Analysis comparing simulated reference populations and sampling locations represented, for samples of white-tailed deer (*Odocoileus virginianus*) from the mid-Atlantic region of the United States. Red circles: simulated populations of wild deer for each cluster inferred with spatial Bayesian clustering algorithm, except Cluster 4 which was represented by only 17 samples and therefore a reference population was not created. Orange circles: counties represented by simulated populations.

**Supplementary Figure S5.** Principal Coordinate Analysis comparing simulated reference populations and sampling locations represented for samples of white-tailed deer (*Odocoileus virginianus*) from the mid-Atlantic region of the United States. Red circles: simulated populations of wild deer for each state. Orange circles: counties represented by simulated populations.

**Supplementary Figure S6.** Principal Coordinate Analysis comparing simulated reference populations and sampling locations represented, for samples of white-tailed deer (*Odocoileus virginianus*) from the mid-Atlantic region of the United States. Red circles: simulated populations of wild deer for each physiographic province. Orange circles: counties represented by simulated populations.
